# A Severe Acute Pancreatitis Mouse Model Transited from Mild Symptoms Induced by a “Two-Hit” Strategy with L-Arginine

**DOI:** 10.3390/life12010126

**Published:** 2022-01-16

**Authors:** Jing Yang, Xujiao Tang, Qingqing Wu, Panpan Ren, Yishu Yan

**Affiliations:** Department of Pharmaceutics, School of Pharmaceutical Sciences, Jiangnan University, Wuxi 214122, China; yangjing@jiangnan.edu.cn (J.Y.); 6201507011@stu.jiangnan.edu.cn (X.T.); 6191502015@stu.jiangnan.edu.cn (Q.W.); 6201507007@stu.jiangnan.edu.cn (P.R.)

**Keywords:** L-arginine, severe acute pancreatitis, two-hit, multi-organ failure, mouse model

## Abstract

To develop a severe acute pancreatitis (SAP) model transited from mild symptoms, we investigated a “two-hit” strategy with L-arginine in mice. The mice were intraperitoneally injected with ice-cold L-arginine (4 g/kg) twice at an interval of 1 h on the first day and subjected to the repeated operation 72 h afterwards. The results showed the “two-hit” strategy resulted in the destructive damage and extensive necrosis of acinar cells in the pancreas compared with the “one-hit” model. Meanwhile, excessive levels of pro-inflammatory mediators, namely IL-6 and TNF-α, were released in the serum. Remarkably, additional deleterious effects on multiple organs were observed, including high intestinal permeability, kidney injury, and severe acute lung injury. Therefore, we confirmed that the SAP animal model triggered by a “two-hit” strategy with L-arginine was successfully established, providing a solid foundation for a deeper understanding of SAP initiation and therapy research to prevent worsening of the disease.

## 1. Introduction

The annual incidence of acute pancreatitis (AP) is estimated at 0.005–0.08% of the total population [1,2]. The majority of cases are mild and self-limiting, with an associated mortality rate of no more than 1% [3]. However, in some cases, the patients who survive the initial AP die following “second hit”-induced severe acute pancreatitis (SAP), which results in a cascade of inflammatory reactions and multiple organ dysfunction. In particular, fatal acute lung injuries eventually result in a mortality rate of up to 10–24% [3,4].

Currently, the primary cause of these symptoms is attributed to the interstitial leakage of pancreatic lipase, resulting in adipose lipolysis and increased levels of unsaturated fatty acids. These toxic fatty acids stimulate the excessive release of inflammatory markers and an inflammatory storm that can drive disease progression with eventual multi-organ failure [5]. Accumulating evidence has revealed that an array of innate immune components, including neutrophils and monocytes/macrophages, participate in the pathogenesis and contribute to disease development [4].

However, the understanding of SAP initiation and development is still very far from complete, and the factors that determine the severity of the disease are unknown due to the complexity of the innate immunity networks [6]. Therefore, treatment methods are limited to conservative supportive care.

During the past few decades, animal models using mice and rats have remained the main approach to deeply understand the pathogenesis of AP. Several models, such as caerulein [7], the intraductal infusion of sodium taurocholate [8,9], L-arginine [10], pancreatic duct obstruction-induced AP [11] and AP evoked by pancreatic ischemia with reperfusion [12], have been developed to meet the various requirements of the research. Indeed, substantial advancements have been made for several types of AP, especially for the diseases of a mild-to-moderate degree, and have yielded several available therapeutic options [13,14,15].

However, these models lack the transition from mild symptoms to the advanced development phase when cytokine storms and multiple organ failure occur. As a result, it is difficult to provide an in-depth elaboration of each of the specific roles of the innate immune components that are involved in the exacerbation of the disease with the current strategies.

L-arginine is a promising choice to induce SAP from mild AP. On the one hand, it selectively damages pancreatic acinar cells and directly leads to necrotizing pancreatitis [16]. On the other hand, it seems that L-arginine has the ability to induce a controllable, dose- and time-dependent pancreatic injury; a higher dose of L-arginine induces AP, whereas low and repeated injections induce chronic pancreatitis [17].

Currently, AP models are induced under the “one-hit” strategy by intraperitoneally injecting L-arginine (4 g/kg each) twice, with each injection administered 1 h apart. With this model, the mice undergo inflammatory peaks 24 h after the L-arginine injection. Mild pancreatic damage is associated with mild pulmonary inflammation that begins to resolve 72 h after L-arginine administration [18]. The mice then undergo the tissue repair and regeneration process, without transitioning to the SAP phase accompanied by cytokine storms and multi-organ failure [19]. Therefore, the model was still limited to the mild or moderate degree of the disease.

Herein, we present a “two-hit” strategy with L-arginine to set up a standardized protocol for an SAP mouse model with multi-organ failure. We intraperitoneally injected the mice with ice-cold L-arginine (4 g/kg) twice at an interval of 1 h on the first day and repeated the operation 72 h afterwards. Then, we confirmed the model was established by systematically comparing the histological and the biomarker changes of AP. The associated intestinal injury, acute lung injury and kidney injury were also investigated. Through these investigations, we found an option for a reproducible SAP model with multi-organ failure.

## 2. Materials and Methods

### 2.1. Materials

L-Arginine (Art. No. JA2774) was purchased from Bomei Biotechnology Co., Ltd. (Hefei, China). The mouse interleukin-6 (IL-6) enzyme-linked immunosorbent assay (ELISA) kit (Art. No. KE10007, sensitivity 3.8 pg/mL, antibody specific for mouse IL-6) and the mouse tumor necrosis factor-α (TNF-α) ELISA kit (Art. No. KE10002, sensitivity 1.0 pg/mL, antibody specific for mouse TNF-α) were from ProteinTech (Wuhan, China). FITC-labeled dextran (Art. No. R-FD-006, Mr 100,000) was purchased from Xi’an Ruixi Biological Technology Co., Ltd. (Xi’an, China). Mouse high mobility group protein 1 (HMGB-1, Art. No. D721089-0096) ELISA kits were purchased from Sangon Biotech (Shanghai, China).The hematoxylin and eosin (H&E) staining kit (Art. No. ab245880) was purchased from Abcam (Cambridge, UK). The anti-HMGB-1 rabbit polyclonal anti-body (Art. No. D160488-0025, reactive to human, mouse and rabbit) was purchased from Sangon Biotech (Shanghai, China).The Alexa Fluor 488-labeled donkey anti-rabbit IgG (Art. No. A423) was purchased from Beyotime (Nanjing, China). Lipase activity assay kits (Art. No. A054-2-1) and α-amylase activity assay kits (Art. No. 100000060) were obtained from Jiancheng Biotech (Nanjing, China).

### 2.2. Animals and Treatment

The Animal Research Committee of Jiangnan University approved all the animal experiments (No. 20210315i0400630 (035)). The issue date was 15 March 2021, and the expiry date is 15 March 2023. In total, fifty-five male mice (8 weeks old) outbred from the Institute of Cancer Research (ICR, 25–30 g) were purchased from Cavens Laboratory Animal Co., Ltd. (Changzhou, China). The mice were divided into the following three groups: the control group (*n* = 5), the “one-hit” model group (*n* = 13) and the “two-hit” model group (*n* = 16). The animals were restricted from eating overnight before the L-arginine injection. Otherwise, they had free access to standard rodent chow and sterilized water during the whole project.

The L-arginine buffer (with a concentration of 8% in PBS buffer, pH = 7.4) was incubated in ice-cold or 37 °C PBS buffer (pH = 7.4). As a pre-experiment, the 37 °C solution was injected intraperitoneally into the mice twice at an interval of one hour, each with a 4 g/kg dose (the final volume was about 1.3 mL, dependent on the weight of each mouse). Meanwhile, the ice-cold solution was used to prepare for the “one-hit” model with the same procedure.

For the “two-hit” model, the mice who underwent the “one-hit” procedures were allowed to recover for 72 h and were then re-administered L-arginine (4 g/kg, pH 7.4) twice at a 1 h interval. The group received identical PBS injections to the “two-hit” model group, which were used as vehicles.

The mice in the “one-hit” and “two-hit” groups were treated with 0.05 mg/kg buprenorphine (0.05 mg/mL) every 12 h for pain control [20]. The final volume of the drug was calculated according to the weight of the mouse and was about 25 μL during each injection. For the survival rate calculation, the mice (*n* = 8) were monitored (the observation lasted for 150 h) immediately after L-arginine was given for the first time, and the survival rate changes were recorded every 2 h. During this period, two mice were subjected to early euthanasia at 105 h and 115 h because of severe emaciation, lack of response to stimuli, no activity, and poor respiration quality with only gasping.

The animals were euthanized before the scheduled time to avoid suffering according to the published standards, including the following: the appearance of the mice, such as piloerection or emaciation; level of consciousness; activity; response to stimulus; eyes being open or closed; respiration rate; and respiration quality [21]. The other mice were sacrificed 24 h after being subjected to the “one-hit” or “two-hit” L-arginine injury. The mice underwent anesthesia by isoflurane inhalation followed by retro-orbital blood collection. About 300 μL blood was extracted from each mouse. Then, they were euthanized by CO_2_ inhalation.

### 2.3. Methods

#### 2.3.1. H&E Staining

The fresh tissues were fixed in 4% paraformaldehyde, embedded with paraffin for hematoxylin and eosin (H&E) staining, and examined using light microscopy. Two investigators who were blind to the experimental treatment scored the degree of tissue injury according to the previously described scoring standards (Table 1, Table 2 and Table 3).

#### 2.3.2. Measurement of Serum Lipase and α-Amylase Activity

The blood from the mice was collected, kept at 4 °C overnight, and centrifuged (4000 rpm, 10 min, 4 °C). The supernatant was collected for further assays. The serum activity of the lipase and α-amylase was detected using the assay kits according to the manufacturer’s instructions. During the lipase activity assay, the lipase catalyzes the hydrolysis of oil esters into fatty acids. The formation rate of fatty acids is then determined by the copper soap method, which is based on the absorbance of a fatty acid–copper complex at 715 nm. During the α-amylase activity assay, the enzyme will cleave the substrate to produce smaller fragments with the ability to form chromophore after I_2_ is added. The product can then be measured based on the absorption at 660 nm.

#### 2.3.3. ELISA of IL-6 and TNF-α

The serum levels of IL-6 and TNF-α were measured with ELISA kits (ProteinTech, Wuhan, China) according to the manufacturer’s protocols.

#### 2.3.4. The Organ Index Measurement

The lungs and pancreas were taken out immediately after the mice were sacrificed. The weight of the organs was recorded. The organ index was calculated as (wet organ weight/body weight) × 100%.

#### 2.3.5. Permeability of the Intestinal Tissue 

After the mice were sacrificed, 5 cm of intestinal tissue near the duodenum was removed [24]. The intestinal lumen was gently washed with a PBS buffer. One side of the intestine was ligated. Then, the intestinal lumen was filled with 200 μL of FITC-dextran and the other side was ligated. The intestinal pouch was gently shaken in the PBS buffer at room temperature for 60 min. The permeability of the intestinal wall was evaluated by measuring the leaked amount of FITC-dextran.

#### 2.3.6. Statistical Analysis

The data were expressed as mean ± SEM. The difference among multiple groups was assessed using one-way variance followed by Bonferroni’s multiple comparison test. The results were considered statistically significant at *p* < 0.05. All analyses were conducted using GraphPad Prism 8 software (GraphPad Software Inc., San Diego, CA, USA). The rationale of the animal number was confirmed by a running power analysis using the G *power 3.1.9.7 software (Dusseldorf, North Rhine-Westphalia, Germany).

## 3. Results 

**Figure 1:**
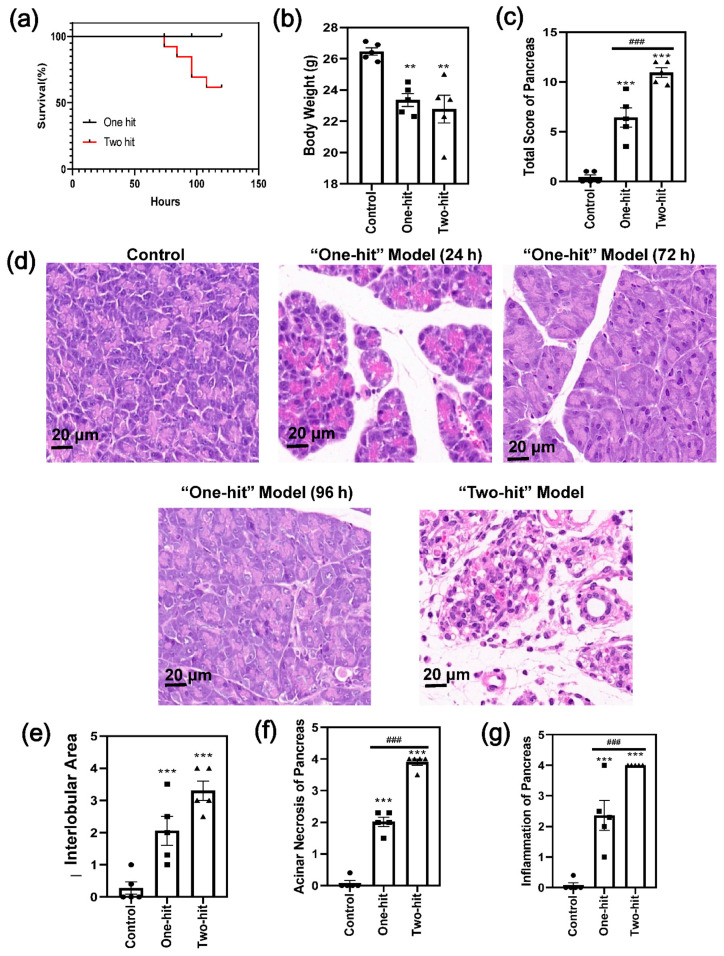
The comparison of the severity of AP between the “one-hit” model and the “two-hit” model in mice. (**a**) Time course survival rate. (**b**) Body weight of the mice after the L-arginine challenge. (**c**) The total histological score of the analysis of the degree of pancreatic damage. (**d**) The representative H&E images of the pancreas with the “one-hit” model (24 h, 72 h, and 96 h after L-arginine injury) and the “two-hit” model (24 h after the second hit). (**e**–**g**) The histological score analysis of the degree of pancreatic damage with regard to (**e**) edema, (**f**) acinar necrosis, and (**g**) inflammation of the pancreas. (* represents the control group vs. the model group, ** *p* < 0.01, *** *p* < 0.001; # represents the “one-hit” group vs. the “two-hit” group, ### *p* < 0.001).

First, we performed primary research to compare the degree of severity between the “one-hit” and “two-hit” model. In the “two-hit” model, 100% of the mice suffered from pancreas damage 24 h after the last dose. The death of the mice happened continually and reached over 30% within a follow-up period of 150 h (Figure 1a). By contrast, the L-arginine solution did not cause any mortality with the “one-hit” model, and 20% of the mice did not show any histological changes in AP. In parallel with this, body weight reduced significantly in the model groups, with no difference between the “one-hit” and “two-hit” operations (Figure 1b).

The histological images of the “one-hit” model were compared with those of the “two-hit” model (Figure 1c,d and Figure S1). The changes were relatively moderate in the “one-hit” model, with cellular swelling, the disruption of the histoarchitecture, neutrophil infiltration, and acinar cell vacuolization and necrosis. In addition, the degree of cellular swelling and the signs of inflammation were reduced at 72 h and 96 h, respectively, compared with 24 h, indicating that the mice recovered from the L-arginine injury. By contrast, 90% of the acinar cells were destructively damaged, and extensive necrosis occurred in the “two-hit” model. Moreover, the interlobular space (Figure 1e and Figure S1), acinar necrosis (Figure 1f), and inflammatory cell infiltration (Figure 1g) were significantly higher according to the histological score analysis [22]

**Figure 2:**
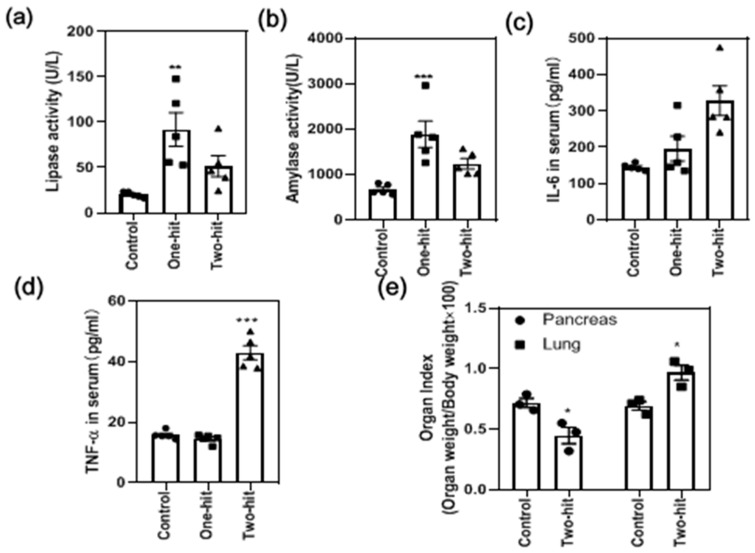
The determination of the biochemical parameters in response to the attack of L-arginine, including (**a**) lipase activity, (**b**) α-amylase activity, (**c**) IL-6 concentration, and (**d**) TNF-α concentration in the serum, as well as the changes in the (**e**) organ index. (* represents the control group vs. the model group, * *p* < 0.05, ** *p* < 0.01, *** *p* < 0.001).

The serum lipase and α-amylase released in the serum increased significantly with the “one-hit” model but decreased significantly after the second hit (Figure 2a,b). A hyper-inflammatory state was also marked by the significant elevation of IL-6 (Figure 2c) and TNF-α with the “two-hit” model (Figure 2d). Meanwhile, the pancreas index (Figure 2e) slightly increased in the “one-hit” model, whereas it decreased dramatically after the second hit. Moreover, the lung index was substantially elevated, demonstrating a pronounced edema formation in the lung tissue (Figure 2e).

**Figure 3:**
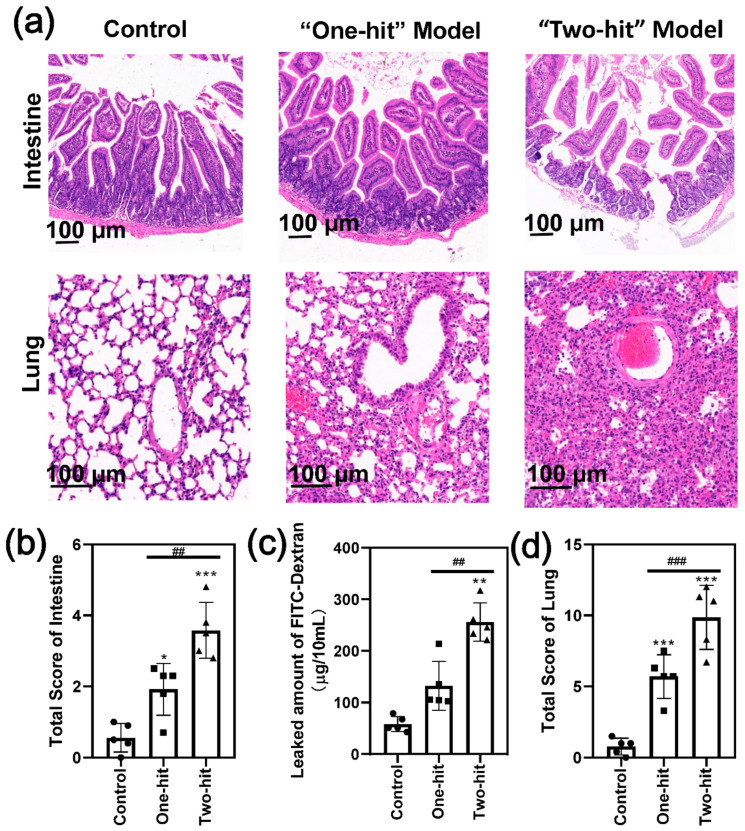
The second hit of L-arginine induced severe intestine and lung injury in the mice. (**a**) The representative H&E images of the intestine and lung with the “one-hit” and “two-hit” models. (**b**) The histological score analysis of the degree of intestinal damage. (**c**) The permeability assay of intestinal tissue in SAP. (**d**) The histological score analysis of the degree of damage to the lung. (* represents the control group vs. the model group, * *p* < 0.05, ** *p* < 0.01, *** *p* < 0.001; # represents the “one-hit” group vs. the “two-hit” group, ## *p* < 0.01, ### *p* < 0.001).

We further compared the histological changes of the jejunum, which is closely linked to the pancreas (Figure 3a). The results showed that the epithelium barrier was relatively complete, and the basement membrane became thick with the “one-hit” model; however, the epithelium was completely separated and the basement membrane collapsed with the “two-hit” model. Consequently, the total intestine score increased by two-fold (Figure 3b) [23]. Consistent with this, the amount of FITC-dextran that passed the intestinal wall was significantly greater than that of the control group, indicating that gut permeability had increased substantially (Figure 3c).

We also evaluated the degree of lung damage by a visual observation of the lung tissue histology (Figure 3a,d). Moderate lung injury was induced in some of the mice after the first-round attack, as demonstrated by the histological observations of alveolar wall thickening and collapsing and the whole-lung permeability of inflammatory cells and red blood cells. However, the second hit initiated more serious acute lung injury symptoms in all of the mice. The alveolar thickness increased significantly (Figure S2a), and considerably more inflammatory cells accumulated in the lung (Figure S2b) with high alveolar congestion (Figure S2c). Consistent with these observations, we found a near-normal kidney architecture with the “one-hit” group, whereas there was obvious tubular dilatation and cast formation in part of the kidney tissue in the “two-hit” group, indicating a more serious tissue injury (Figure S3). These results indicated that the organ injury developed to a very high level, confirming that the modified model induced homogeneous SAP in mice.

## 4. Discussion

SAP is the most frequent digestive disease leading to death in clinics, and it is related to a second hit of AP [25]. Currently, the understanding of SAP etiology is still limited due to the lack of proper disease models developed from AP. For example, caerulein-induced pancreatitis is the most widely used AP animal model. However, after a first-time injury, each subsequent infusion of caerulein causes significantly less pronounced destruction of the pancreatic tissue [26]. After a few days, the exocrine pancreas is regenerated and restored to normal. On the other hand, the administration of capsaicin after caerulein results in calcitonin gene-related peptide (CGRP) being released from unmyelinated, activating, capsaicin-sensitive sensory nerves. This event may produce neurogenic inflammation, eventually leading to the aggravation of pancreatic damage and chronic pancreatitis, without SAP development [27,28].

To facilitate the investigation of the initial factors that trigger SAP, we developed an SAP model transited from mild symptoms with L-arginine using the “two-hit” strategy in mice. Ice-cold L-arginine was repeatedly injected intraperitoneally 72 h after the “one-hit” operation. Indeed, the H&E images showed destructive damage to the acinar cells and the presence of extensive necrosis after the second hit, indicating that the model was successfully established.

The serum lipase and α-amylase released in the serum are two important parameters regarding pancreas activity [29]. However, their amount increased in the “one-hit” model and deceased in the “two-hit” model. We speculated that this could be attributed to the necrotic pancreatic acinar cells losing their enzyme secretory function in the “two-hit” model. Indeed, the pancreas index increased after the first hit, indicating high tissue edema in response to the L-arginine, and decreased substantially after the second hit, indicating the pancreas tissue experienced more serious destruction and a self-digestion process in the “two-hit” model [30]. These data clearly showed that the “two-hit” model augmented the injuries of the “one-hit” model.

Further, we found the disease rapidly progressed into a highly inflammatory condition with multiple complications after the second hit. The serum inflammatory levels increased significantly. By comparing the “one-hit” model with the “two hit” model, we could study the exact roles of the innate immune cells and the pro-inflammatory cytokines in the onset and progression of SAP. For example, significantly elevated serum IL-6 and TNF-α were found with the “two-hit” model, indicating that they might be the key factors promoting the aggravation of inflammation. Indeed, they are promising biomarkers to predict the severity of the disease in clinics, as they are closely related to the rates of mortality and acute respiratory distress syndrome [31]. The roles of other cells and cytokines in the transition from mild symptoms to SAP have now been investigated in subsequent studies with the current models.

Intestinal homeostasis is essential during the management of SAP. Accumulating studies have confirmed that intestinal barrier dysfunction, increased gut permeability, and bacterial translocation initiate the subsequent multiple organ dysfunction syndrome [32,33]. Our results confirmed that a higher intestinal permeability was induced by the second injury.

More seriously, the destruction of the intestinal tissue leads to kidney injury and severe acute lung injury, which is the leading cause of mortality. As a result, the second hit exacerbated the “one-hit” inflammatory reactions, leading to multiple organ failures with over 30% mortality. This “two-hit” model mimics the pathological process of SAP in humans, where a challenge following a previous stimulation induces an uncontrollable inflammatory response and death.

During the pre-experiments, we found no pancreas damage when the solution of L-arginine (4 g/kg, 8%) reached 37 °C through histological observation with the “one-hit” model. In contrast, most of the mice treated with ice-cold L-arginine were induced with AP successfully. A previous study revealed that L-arginine forms two to three molecule aggregates at low temperature [34]. Moreover, it is well recognized that poly-L-arginine induces cell membrane damage among different cell types through the charge effect [35]. We supposed that the aggregate of L-arginine caused a similar cellular damage effect by substantially increasing positive electrical charges. In the following experiments, we continually used the ice-cold L-arginine to ensure the establishment of the AP model.

The limitation of the model lies in the difference in pathophysiology compared to human AP. Different from the natural acinar toxins, such as alcohol and lipopolysaccharide, L-arginine has a clinically irrelevant initiating mechanism that selectively damages the pancreatic acinar cells, thereby resulting in a different distribution of parenchymal injury. Secondly, L-arginine triggered the acinar cell necrotization directly, whereas a series of earlier cellular events, including premature trypsinogen activation, endoplasmic reticulum stress, and impaired autophagy, were skipped [2]. Therefore, this “two-hit” model resembles the cytokine storm in the deteriorated phase for humans, which is complementary with other models for the early phase of the disease.

## 5. Conclusions

In summary, we presented a “two-hit” strategy with L-arginine to induce an SAP mouse model. The results showed the disease developed into a new phase after the second hit of L-arginine, with a high intestinal permeability and severe acute lung injury. Therefore, this model can successfully link local events to systematic inflammation, and opens the door for SAP pathology and therapy research along with the study of multi-organ failure.

## Figures and Tables

**Table 1:** The criteria for the evaluation of pancreatic injury [22].

Score	Edema	Acinar Necrosis	Inflammation
0	Absent	Absent	0–5 leukocytes/HPF
1	Diffuse expansion of the interlobar septae	1–4 necrotic cells/(High power field, HPF)	6–15 leukocytes/HPF
2	Diffuse expansion of the interlobubar septae	5–10 necrotic cells/HPF	16–25 leukocytes/HPF
3	Diffuse expansion of the interacinar septae	11–16 necrotic cells/HPF (foci of confluent necrosis)	26–35 leukocytes/HPF
4	Diffuse expansion of the intercellular spaces	>16 necrotic cells/HPF (extensive confluent necrosis)	>36 leukocytes/HPF

**Table 2:** The criteria for the evaluation of intestinal injury [23].

Score	
0	Normal mucosal villi.
1	Development of subepithelial Gruenhagen’s space, usually at the apex of the villus; often with capillary congestion.
2	Extension of the subepithelial space with moderate lifting of the epithelial layer from the lamina propria.
3	Massive epithelial lifting down the sides of villi. A few tips may be denuded.
4	Denuded villi with lamina propria and dilated capillaries exposed. Increased cellularity of the lamina propria may be noted.
5	Digestion and disintegration of the lamina propria; hemorrhage and ulceration.

**Table 3:** The criteria for the evaluation of lung injury [22].

Score	Thickness of the Alveolar	Infiltration of the Neutrophils	Alveolar Congestion
0	Absent	Absent	Absent
1	Discrete	Discrete	Small foci
2	Moderate	Moderate	Large foci
3	Severe	Severe	Diffuse

## Data Availability

Data available on request from the authors.

## Supplementary Materials

The following supporting information can be downloaded at: https://www.mdpi.com/article/10.3390/life12010126/s1, Figure S1: The H&E images of the pancreatic tissue after injury; Figure S2: The evaluation criteria of the lung tissue; Figure S3: The H&E images of the kidney tissue after injury.
