# Supplementary material for: A Severe Acute Pancreatitis Mouse Model Transited from Mild Symptoms Induced by a “Two-Hit” Strategy with L-Arginine
Source: Life (Basel). 2022 Jan 16;12(1):126. doi: 10.3390/life12010126 (PMC8779052; doi:10.3390/life12010126)
Supplement: Supplementary file 1 [file life-12-00126-s001.zip › life-1556521-supplementary.pdf]

The Supplementary Materials for

A Severe Acute Pancreatitis Mouse Model Transited from Mild Symptoms Induced by a “Two-Hit” Strategy with L-Arginine

Jing Yang, Xujiao Tang, Qingqing Wu, Panpan Ren and Yishu Yan

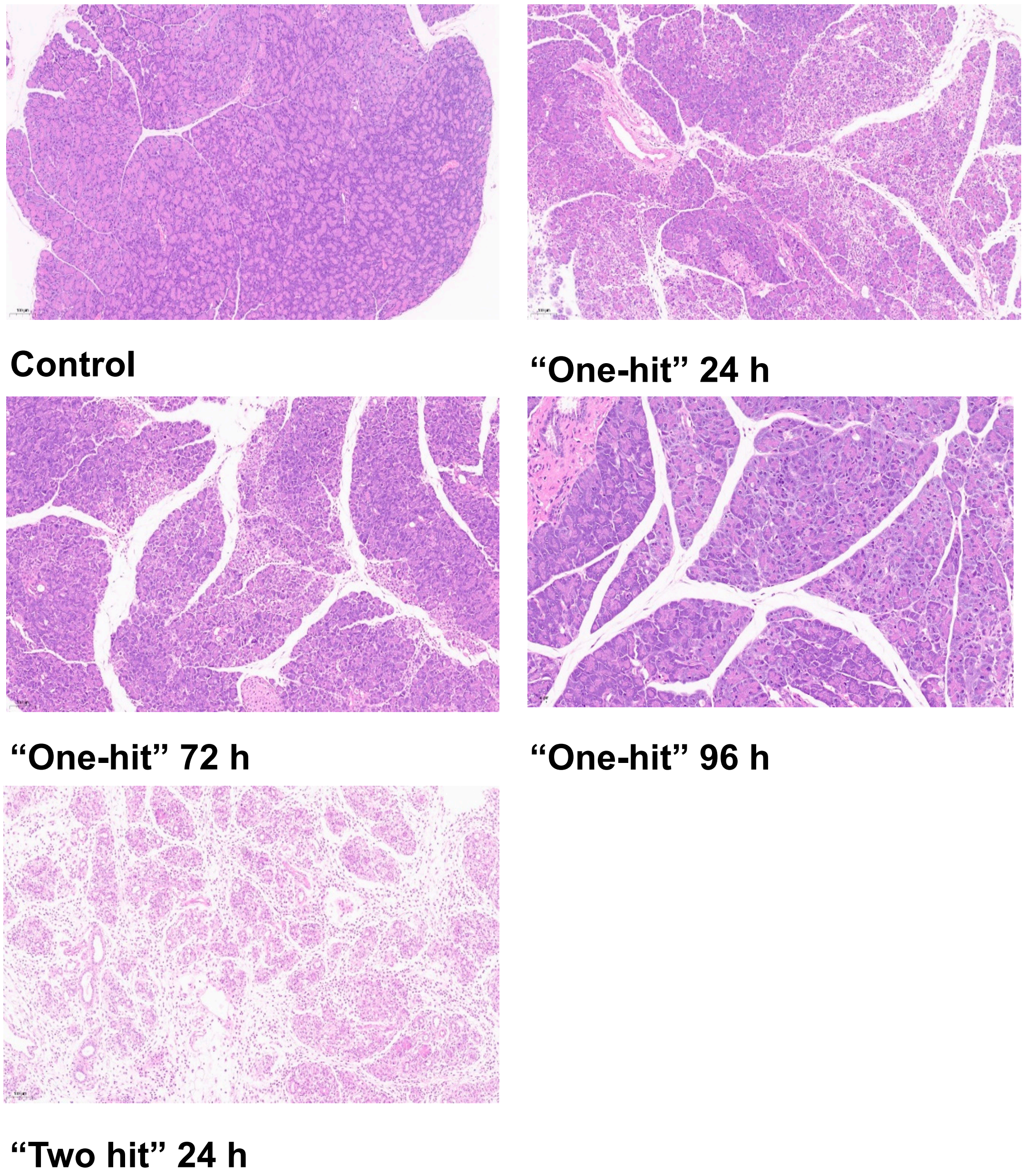

Figure S1. The H&E images of the pancreatic tissue after injury.

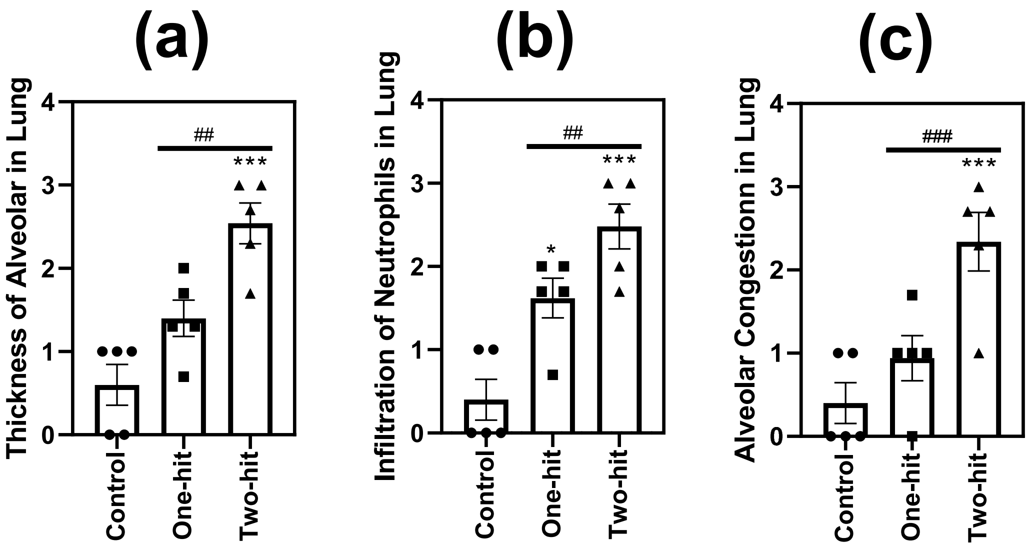

Figure S2. The evaluation criteria of the lung tissue.

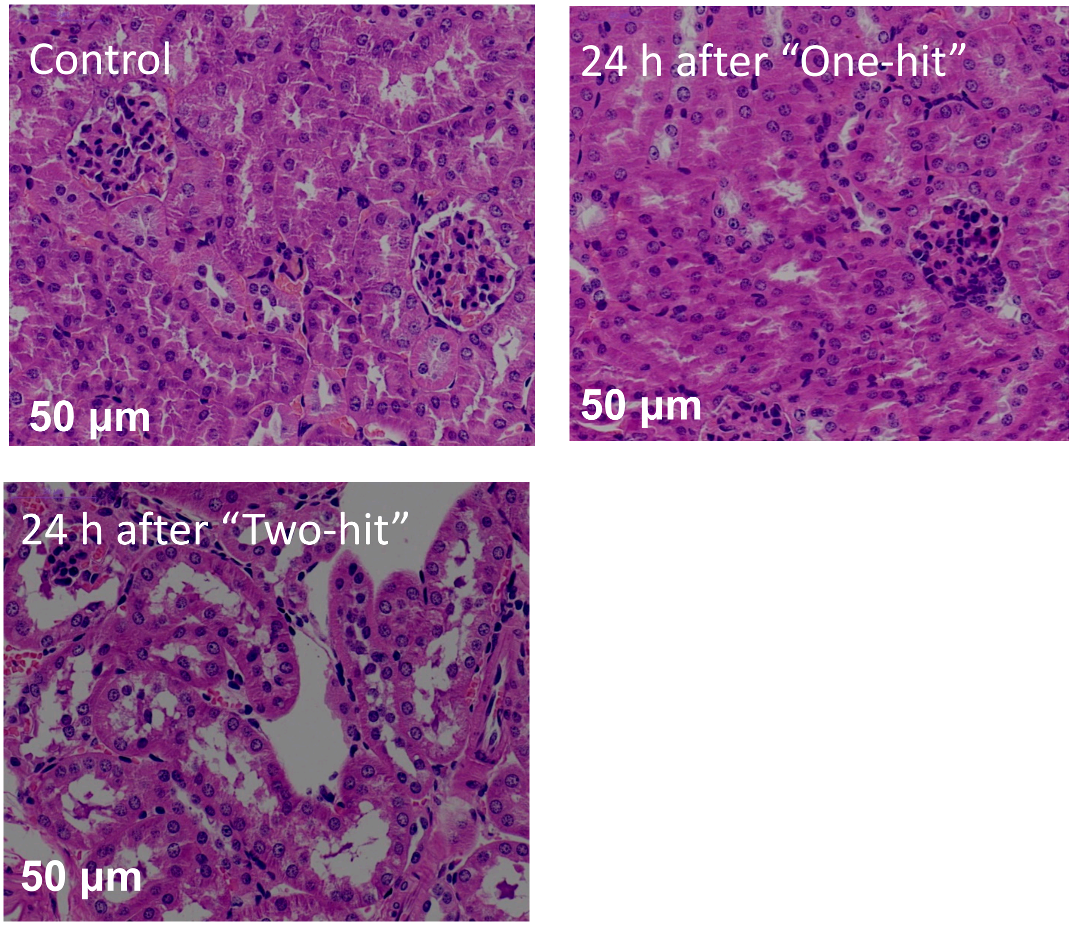

Figure S3. The H&E images of the kidney tissue after injury.
